# Antioxidant capacity of N-acetylcysteine against the molecular and cytotoxic implications of cadmium chloride leading to hepatotoxicity and vital progression

**DOI:** 10.1007/s11356-022-23823-x

**Published:** 2022-11-02

**Authors:** Rasha S. Albeltagy, Shauq M. Dawood, Farah Mumtaz, Ahmed E. Abdel Moneim, Ola H. El-Habit

**Affiliations:** 1 Department of Zoology and Entomology, Faculty of Science, Helwan University, Cairo, Egypt; 2 Department of Biochemistry, College of Science, Osmania University, Telangana State, Hyderabad, India

**Keywords:** Cadmium chloride, Carcinogenesis, Bcl2/Bax ratio, Cell cycle, Oxidative stress

## Abstract

Many studies have reported that cadmium (Cd) can induce liver cell injury; however, the toxicity mechanisms of Cd on the liver have not been fully explained. Thirty-two male albino rats were divided into four groups: the control group, the N-acetylcysteine (NAC) group orally as effervescent instant sachets with a concentration of 200 mg dissolved in distilled water and dosage was 200 mg/kg body weight freshly prepared, the cadmium chloride (CdCl_2_) group (treated with 3 mg/kg orally), and the N-acetylcysteine (NAC) + cadmium chloride group (treated with 200 mg/kg orally post to CdCl_2_) for 60 days. The NAC alone did not make notable changes in most of the parameters. The CdCl_2_ alone, compared to control, induced significant alterations in oxidative stress markers (increment in lipid peroxidation (LPO) and nitric oxide (NO)) and antioxidant defense system (decrement in superoxide dismutase (SOD), catalase (CAT), glutathione (GSH), and glutathione peroxidase (GPx)), which resulted in a downregulation of pro-apoptotic Bcl2-associated X protein (Bax) and caspase-3 and upregulation of anti-apoptotic B-cell leukemia/lymphoma 2 (Bcl2) protein as well as the survival fate of hepatic cells. Post-administration of NAC to CdCl_2_ resulted in a reduction in oxidative stress markers, shifting of cells from the *G*_2_/*M* phase to the *G*_0_/*G*_1_ inhibiting signal-regulated kinase activation, and impairment of the anti-apoptotic signaling pathway when compared to the CdCl_2_ group alone. Accordingly, the Bcl2/Bax ratio was reduced to 1.17-fold change, as an adaptive process to hepatic tissue injury. These findings demonstrated that NAC would attenuate the possibility of oxidative stress and cytotoxicity of hepatic tissue induced by CdCl_2_.

**Supplementary Information:**

The online version contains supplementary material available at 10.1007/s11356-022-23823-x.

## Introduction

Cadmium (Cd) generally exists as a divalent cation, complexed with other elements (e.g., CdCl_2_) (Bernhoft 2013; Liu et al. 2019). The main sources of occupational cadmium exposure include fume inhalation, electroplating, nickel–cadmium battery industry, PVC, plastics, and paint pigments (Al-Brakati et al. 2021; Dkhil et al. 2020). Moreover, it can be found in soil, due to cadmium-containing insecticides, fungicides, and commercial fertilizers (Olszowski et al. 2012; Sulaiman et al. 2020).

Cadmium toxicity causes severe damage in the gene expression process. The disruption results in creating reactive oxygen species, disrupting the oxidative stress, causing apoptosis, and halting the DNA repair process. These results contribute to carcinogenesis (Hartwig 2013; Waalkes 2003; Đukić-Ćosić et al. 2020; Albeltagy et al. 2021). One significant result is apoptosis, whereby cells are exposed to external stresses, causing programmed cell death (Gu et al. 2018; Cardinale et al. 2010). Another indication of apoptosis, showing damage in the pathways of the affected cells, would be the imbalance of the pro-apoptotic and anti-apoptotic (Zawlik et al. 2005). A measuring tool is used post an apoptotic stimulus, which is the ratio of Bax/Bcl-2, to determine the mortality fate of the cell (Mahdavi et al. 2018).

The oxidative stress and lipid peroxidation involved in the pathogenesis of various diseases and consequently the role of free radical scavenging antioxidants in organ toxicity and carcinogenicity received much attention (Omata et al. 2010). On the other hand, a recent focus involves understanding the role of natural products with antioxidant activity to improve the hazardous effects of drugs and xenobiotics.

N-acetylcysteine (NAC) has been used as an antioxidant in several in vivo and in vitro studies and recorded antioxidant and radical scavenging activities (Ates et al. 2008). The molecular mechanism through which NAC exerts this activity has been discussed. In this regard, the mechanisms of NAC antioxidant activity may be directed toward oxidant species or indirect because of its ability to act as a precursor of l-cysteine, the building block in glutathione synthesis, and breaking disulfides to restore thiol pools (Mahmoud et al. 2019). NAC treatment offers protection against oxidative stress induced by different toxicants due to its dual role as a nucleophile and as a thiol donor (Suke et al. 2008). Additionally, NAC prevents cell death pathways and inflammation signaling in vital organs by elevating endogenous levels of glutathione and restraining mitochondrial membrane depolarization (Zafarullah et al. 2003; Abdel-Daim et al. 2019) with antifibrotic action (Salamon et al. 2019).

This study aimed to investigate the relevant mechanisms and pathways of Cd carcinogenicity by assessing the cell cycle progression, the Bax/Bcl-2 balance impairment, oxidative stress and histopathological changes of hepatic tissues, and the N-acetylcysteine direct antioxidant property and indirectly as a precursor in glutathione synthesis to mitigate the associated oxidative incidence.

## Material and methods

### Chemicals

Cadmium chloride (CdCl_2_; CAT Number 10043–52-4) was purchased from Sigma-Aldrich Chemical Co. (St. Louis, MO, USA). NAC was purchased from SEDICO Pharmaceutical Co. (Giza, Egypt).

### Experimental animals

Thirty-two male Wistar rats weighing 150–200 g (2–3 months) were purchased from the Faculty of Medicine, Medical Ain Shams Research Institute (MASRI), Cairo, Egypt. Animals were housed in polycarbonate boxes with steel wire tops, bedded with wood shavings. Ambient temperature was controlled at 22 ± 3 °C with relative humidity. Food and water were provided ad libitum. All animals were acclimatized for 1 week before the beginning of the experiment. Animal care and use followed the guidelines of Investigations & Ethics for Laboratory Animal Care at the Department of Zoology, Faculty of Science, Helwan University (approval no. HU2018/Z/FM1118-03).

Animals were divided into 4 groups (8 rats/each): the control group, received 10 ml/kg physiological saline (0.9% NaCl); the NAC group orally as effervescent instant sachets with a concentration of 200 mg dissolved in distilled water and dosage was 100 mg/kg body weight freshly prepared (Mahmoud et al. 2019); the CdCl_2_ group, received 3 mg/kg body weight CdCl_2_ according to Babaknejad et al. (2015); and the NAC + CdCl_2_ group, received 100 mg/kg NAC (Mahmoud et al. 2019) 1 h post to CdCl_2_ for 60 consecutive days.

At the end of the experiment, rats were sacrificed by cervical decapitation and dissected for obtaining the desired tissue. Blood samples were collected immediately from the inferior vena cava and left to clot in dry test tubes and then centrifuged at 3000 × *g* for 10 min to obtain serum. The obtained serum was stored at − 80 °C for further biochemical assays. The liver tissues were collected, a part was fixed in 10% buffered formalin for histopathological investigations, and another part was kept at − 80 °C for oxidative stress markers; ELISA technique and flow cytometry assay were also performed.

### Quantitative real-time PCR analysis

RNA was isolated from freshly removed liver tissue using the TRIzol reagent (Qiagen, Germantown, MD, USA) following the instructions of the manufacturer. The RNA concentration was determined using NanoDrop, and then it was reverse transcribed into cDNA by using a kit of RevertAid™ H Minus Reverse Transcriptase supplied by Fermentas, Thermo Fisher Scientific Inc., Canada, according to the manufacturer’s protocol. An SYBR Green PCR kit (Qiagen, Germany) was used to determine mRNA levels of *Bcl2*, *Bax*, and *Casp3*. Quantitative PCR was performed in triplicate on a ViiA™ 7 PCR system (Applied Biosystems, USA). The relative levels of mRNA were calculated by the 2^−ΔΔCt^ method, which was normalized to the mRNA level of the *Gapdh* housekeeping gene. Primer sequences are shown in Table 1.

**Table 1:** Primer sequences of genes analyzed in real-time PCR

Gene	Primer sequences	Accession number
*Bcl2*	F. 5 ′ CTGGTGGACAACATCGCTCTG R. 5 ′ GGTCTGCTGACCTCACTTGTG	NM_016993.2
*Bax*	F. 5 ′ GGCGAATTGGCGATGAACTG R. 5 ′ ATGGTTCTGATCAGCTCGGG	NM_017059.2
*Casp3*	F. 5 ′ GAGCTTGGAACGGTACGCTA R. 5′ CCGTACCAGAGCGAGATGAC	NM_001284409.1
*Gapdh*	F. 5 ' TCACCACCATGGAGAAGGC R. 5 ′ GCTAAGCAGTTGGTGGTGCA	NM_001289726.1

### Determination of cell cycle phases and apoptosis by flow cytometry

Liver cells (2 × 10^6^) were resuspended in 1 ml ice-cold PBS and then permeated with 70% ice-cold ethanol at 4 °C for 24–48 h. Cells were washed twice by adding 2 ml cold PBS (1800 × *g*, 5 min). The cell pellets were resuspended in 300–500 µl PI/Triton X-100 staining solution (1000 µl of 0.1% Triton + 40 µl PI + 20 µl RNAse) and were incubated at 37 °C for 15 min. The cells were acquired using a BD FACS Canto II flow cytometry (BD Biosciences, USA), and the data were analyzed by BD FACS Diva software.

### Estimation of apoptotic/anti-apoptotic markers by ELISA

Hepatic levels of B-cell lymphoma 2 (Bcl-2) (catalog number NBP2-69,947), Bcl-2-associated X protein (Bax) (catalog number NBP2-69,938), and caspase-3 (catalog number NBP2-75,024) were detected in liver homogenate using the ELISA kit (Abcam Company, Cambridge, UK) following the manufacturer’s instructions.

### Biochemical analysis

#### Determination of liver function

Transaminases (alanine aminotransferase (ALT) and aspartate aminotransferase (AST)) in serum samples were determined according to Reitman and Frankel (1957) while serum alkaline phosphatase (ALP) activity was assayed according to the method described by Belfield and Goldberg (1971).

#### Determination of oxidative stress markers

Grinding and homogenizing of the liver was performed by mixing with 10 mM phosphate buffer (pH 7.4). The supernatant was prepared from the liver by centrifugation of homogenate for 10 min (3000 × *g*) at 4 °C. The lipid peroxide (LPO) level was measured as described by Ohkawa et al. (1979). Nitric oxide (NO) level was detected according to the method of Green et al. (1982) that used the Griess reagent. The activity of reduced glutathione (GSH) was investigated using the method of Ellman (1959). The superoxide dismutase (SOD) level was evaluated following the standard technique described by Nishikimi et al. (1972). The catalase (CAT) activity was demonstrated following the method described by Aebi (1984). The activity of glutathione peroxidase (GPx) was determined according to the procedure described by Paglia and Valentine (1967).

### Histological assay

Tissue samples were fixed in 10% neutral-buffered formalin for 24 h at room temperature (25 °C ± 2). They were dehydrated, embedded in paraffin, sectioned (4–5 μm), and stained regularly with hematoxylin and eosin for histological examination. Furthermore, the alterations of the histopathological parameters of the liver were graded as follows: no: ( −), mild: ( +), moderate: (+ +), and severe: (+ + +) histological alterations, respectively. Ten random fields at 400 × were examined.

### Statistical analysis

Data were presented as mean ± standard deviation (SD). Statistical analysis was conducted using one-way analysis of variance (ANOVA) using SPSS (Statistical Package for the Social Sciences), version 17, followed by Duncan multiple range test (DMRT) post hoc test for the least significant difference between group *P* < 0.05. Furthermore, Pearson’s correlation coefficient (*r*) test was used for correlating data.

## Results

### Results of qRT-PCR

**Fig. 1:**
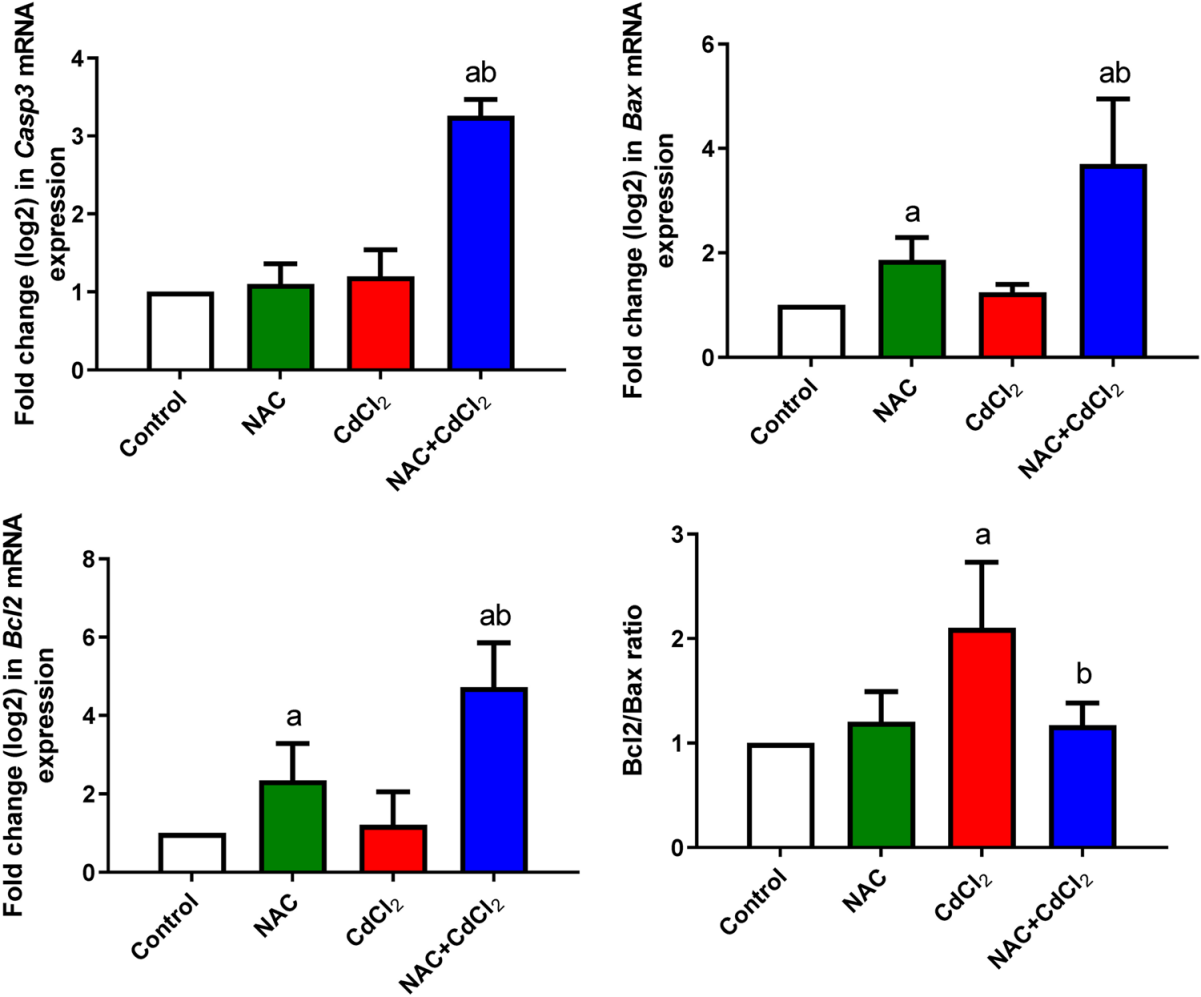
The mRNA expression levels for *Casp3*, *Bax*, and *Bcl2* and *Bcl2/Bax* ratio. Data are expressed as mean ± SD. The relative expression is referenced to *Gapdh* (internal control) and represented as fold change (log2 scale). **a**: Significant change at *P* < 0.05 against the control group. **b**: Significant change at *P* < 0.05 against the CdCl_2_ group

Figure 1 represents the level of *Casp3*, *Bax*, and *Bcl2* expressions in the liver tissue of rats in different groups. The data indicated a non-significant change in *Casp3*, *Bax*, and *Bcl2* expressions in the NAC group (0.76-, 1.50-, and 1.25-fold changes), respectively, compared to the control group. Markedly, there was a significant upregulation in *Bcl2* (1.54-fold change) and *Bcl2/Bax* (2.10-fold changes) and a non-significant change in *Casp3* (1.22-fold change) and *Bax* (0.79-fold change) expression level after a daily treatment with CdCl_2_ compared to their levels in the control group. Post-administration of NAC to CdCl_2_ daily resulted in a significant upregulation in *Casp3* level (3.18-fold change) and *Bax* (2.61-fold change) when compared to both control and CdCl_2_ groups and a significant downregulation in *Bcl2* (0.92-fold change) and *Bcl2/Bax* (1.17-fold change) when compared to the CdCl_2_ group.

### Results of apoptotic/anti-apoptotic markers by ELISA

**Fig. 2:**
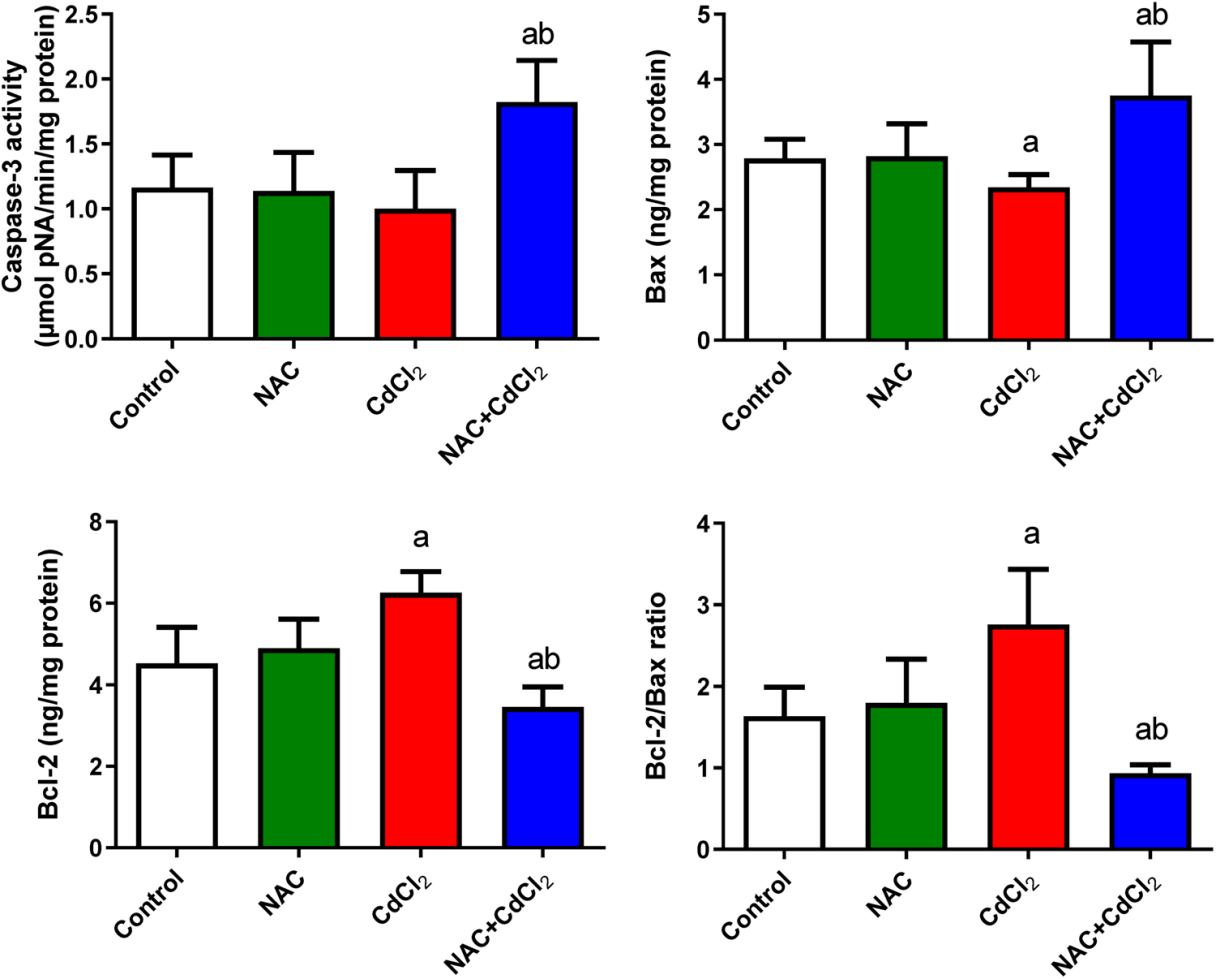
The protein levels of caspase-3, Bax, Bcl2, and Bcl2/Bax ratio in liver tissue of adult male albino rats. Data are expressed as mean ± SD. **a**: Significant change at *P* < 0.05 against the control group. **b**: Significant change at *P* < 0.05 against the CdCl_2_ group

The data indicated that NAC administration showed a non-significant change in apoptotic/anti-apoptotic marker. The CdCl_2_ group showed a significant increase in Bcl2 protein level when compared to its level in the control group, whereas post-administration of NAC to CdCl_2_ showed a significant decrease (*P* < 0.05) in Bcl2 protein level when compared to its level in both control and CdCl_2_ groups. There was a significant decrease in the protein level of Bax and caspase-3 in the CdCl_2_ group compared to the control group, whereas post-treatment of NAC to CdCl_2_ showed a significant increase (*P* < 0.05) in both Bax and caspase-3 when compared to its level in the CdCl_2_ group and the control group. The Bcl2/Bax ratio showed a significant increase in the CdCl_2_ group when compared to the control group, whereas the NAC + CdCl_2_ group showed a significant decrease (*P* < 0.05) in Bcl2/Bax ratio when compared to the control and CdCl_2_ groups (Fig. 2).

### Results of flow cytometry

**Fig. 3:**
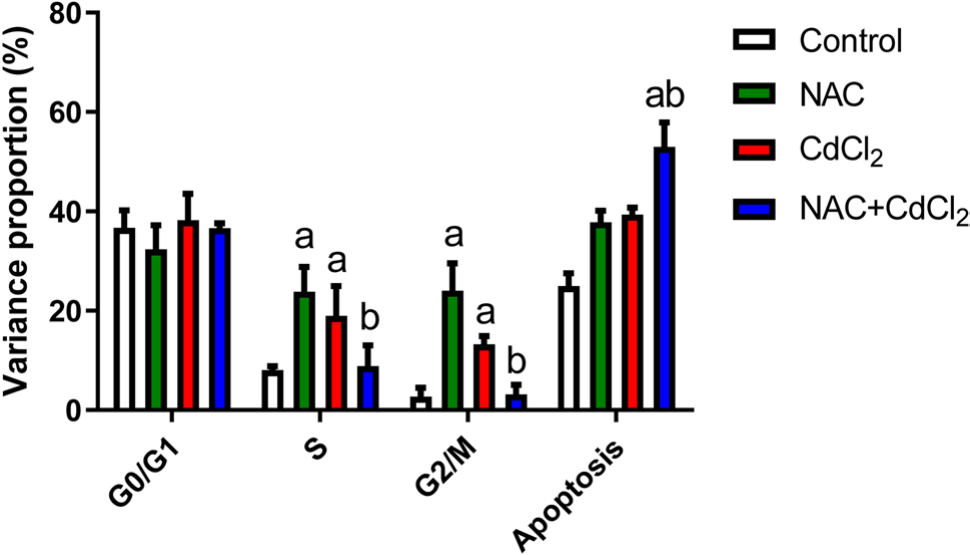
Quantitative analysis of hepatocytes for cell cycle phases and apoptotic percentages. Data are reported as mean ± SD. **a**: Significant change at *P* < 0.05 against the control group. **b**: Significant change at *P* < 0.05 against the CdCl_2_ group

Figure 3 shows the flow analysis data of hepatocytes. NAC administration induced a significant change in both *S* and *G*_2_/*M* percentages when compared to the control values, while the number of cells in *G*_0_/*G*_1_ phase fraction was statistically non-significant in the CdCl_2_ group compared to the control group. However, the number of cells in *S* and *G*_2_/*M* phases increased significantly (*P* < 0.05) when compared to the control group. Interestingly, post-administration of NAC to CdCl_2_ showed a significant decrease in cell numbers in *S* and *G*_2_/*M* phases when compared to the CdCl_2_ group. Moreover, the number of cells at *G*_0_/*G*_1_ was non-significant when compared to the CdCl_2_ group.

### Results of biochemical analysis

#### Oxidative stress markers

**Fig. 4:**
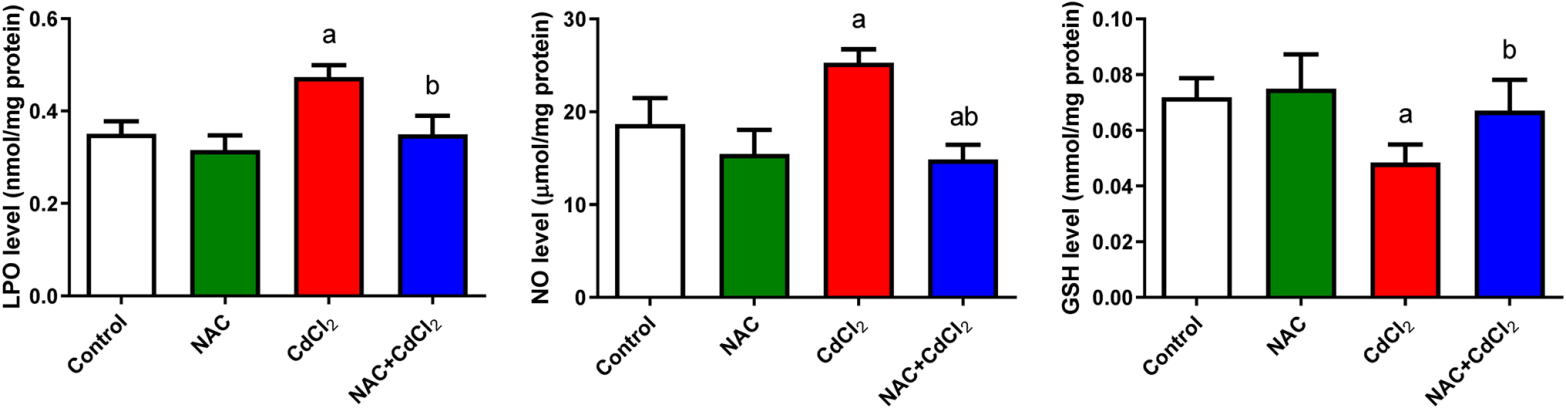
The effect of NAC and/or CdCl_2_ administered on LPO, NO, and GSH levels in liver tissue of adult male albino rats. Data are expressed as mean ± SD. **a**: Significant change at *P* < 0.05 against the control group. **b**: Significant change at *P* < 0.05 against the CdCl_2_ group

**Fig. 5:**
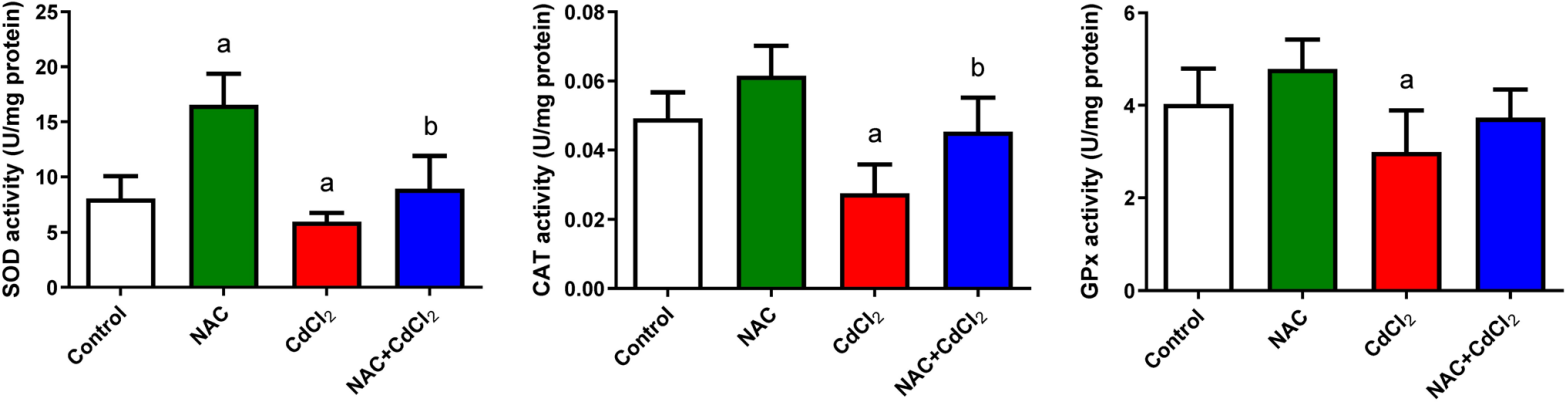
The effect of NAC and/or CdCl_2_ administered on SOD, CAT, and GPx activities in liver tissue of adult male albino rats. Data are expressed as mean ± SD. **a**: Significant change at *P* < 0.05 against the control group. **b**: Significant change at *P* < 0.05 against the CdCl_2_ group

Figures 4 and 5 show the levels of LPO, NO, enzymatic antioxidants, GSH, non-enzymatic antioxidants, and oxidative markers SOD, CAT, and GPx. NAC administration showed a non-significant change in LPO, NO, and GSH and a significant decrease in SOD, CAT, and GPx when compared with the control group. CdCl_2_ induced oxidative stress, as evidenced by a significant (*P* < 0.05) elevation in LPO and NO levels. Data reported a significant decrease (*P* < 0.05) in both GSH content and activities of SOD, CAT, and GPx when compared with the control group. On the other hand, the NAC + CdCl_2_ group indicated an antioxidant potential of NAC. The levels of LPO and NO decreased significantly (*P* < 0.05) when compared to the CdCl_2_ group. Meanwhile, a significant increase (*P* < 0.05) was detected in the activities of SOD, CAT, GPx, and GSH.

#### Liver function enzymes

**Fig. 6:**
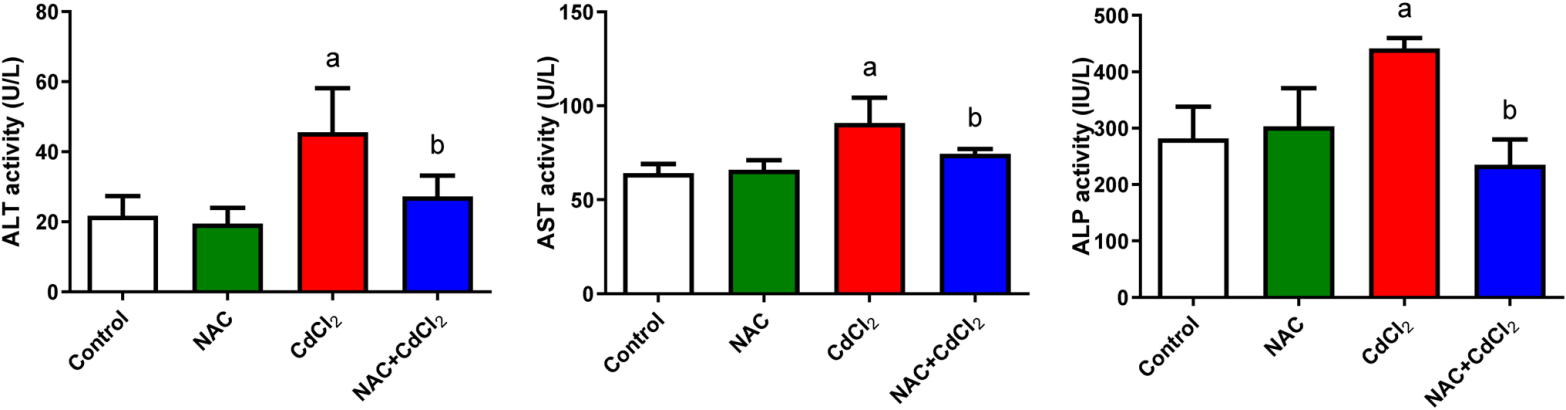
The effect of NAC and/or CdCl_2_ administered on ALT, AST, and ALP activities in the serum of adult male albino rats. Data are expressed as mean ± SD. **a**: Significant change at *P* < 0.05 against the control group. **b**: Significant change at *P* < 0.05 against the CdCl_2_ group

As shown in Fig. 6, NAC administration caused a non-significant change in ALT, AST, and ALP levels in the serum of the group compared to the control group; CdCl_2_ administration caused an elevation in ALT, AST, and ALP levels significantly (*P* < 0.05) in the serum of the group compared to the control group. On the other hand, post-administration of NAC to CdCl_2_ resulted in a significant decrease (*P* < 0.05) in AST and ALP significantly when compared to the CdCl_2_ group.

Pearson’s correlation among different parameters had been tested during this experiment. Pro-apoptotic markers (Cas-3 and Bax) were found to be positively correlated with enzymatic and non-enzymatic molecules and negatively correlated with an anti-apoptotic marker (Bcl-2), oxidative stress markers (LPO and NO), and liver function parameters (ALT, AST, and ALP), while Bcl-2 and liver function parameters were found to be negatively correlated with enzymatic and non-enzymatic molecules and positively correlated with oxidative stress markers. Furthermore, enzymatic and non-enzymatic molecules (SOD, CAT, GPx, and GSH) were found to be positively correlated with pro-apoptotic markers and negatively correlated with Bcl-2, oxidative stress markers, and liver function parameters as indicated in Table 2.

**Table 2:** Pearson’s correlation coefficient among all tested parameters in liver tissue under cadmium chloride (CdCl_2_) stress with prior administration of N-acetylcysteine (NAC)

	Cas-3	Bax	Bcl-2	ALT	AST	ALP	LPO	NO	GSH	SOD	CAT
Bax	.653*										
Bcl-2	− .568	− .646*									
ALT	− .086	− .326	.515								
AST	− .071	− .340	.567	.941**							
ALP	− .665*	− .678*	.795**	.610*	.578*						
LPO	− .335	− .243	.675*	.836**	.843**	.651*					
NO	− .267	− .623*	.574	.847**	.873**	.612*	.654*				
GSH	.216	.408	− .594*	− .823**	− .786**	− .773**	− .770**	− .632*			
SOD	− .021	.050	.065	− .536	− .578*	− .109	− .425	− .620*	.254		
CAT	.106	.257	− .388	− .753**	− .823**	− .452	− .602*	− .763**	.628*	.585*	
GPx	.138	.168	− .158	− .521	− .585*	− .355	− .562	− .466	.589*	.650*	.403

^*^Correlation is significant at the 0.05 level (2-tailed)

^**^Correlation is significant at the 0.01 level (2-tailed)

### Results of histological examination

**Fig. 7:**
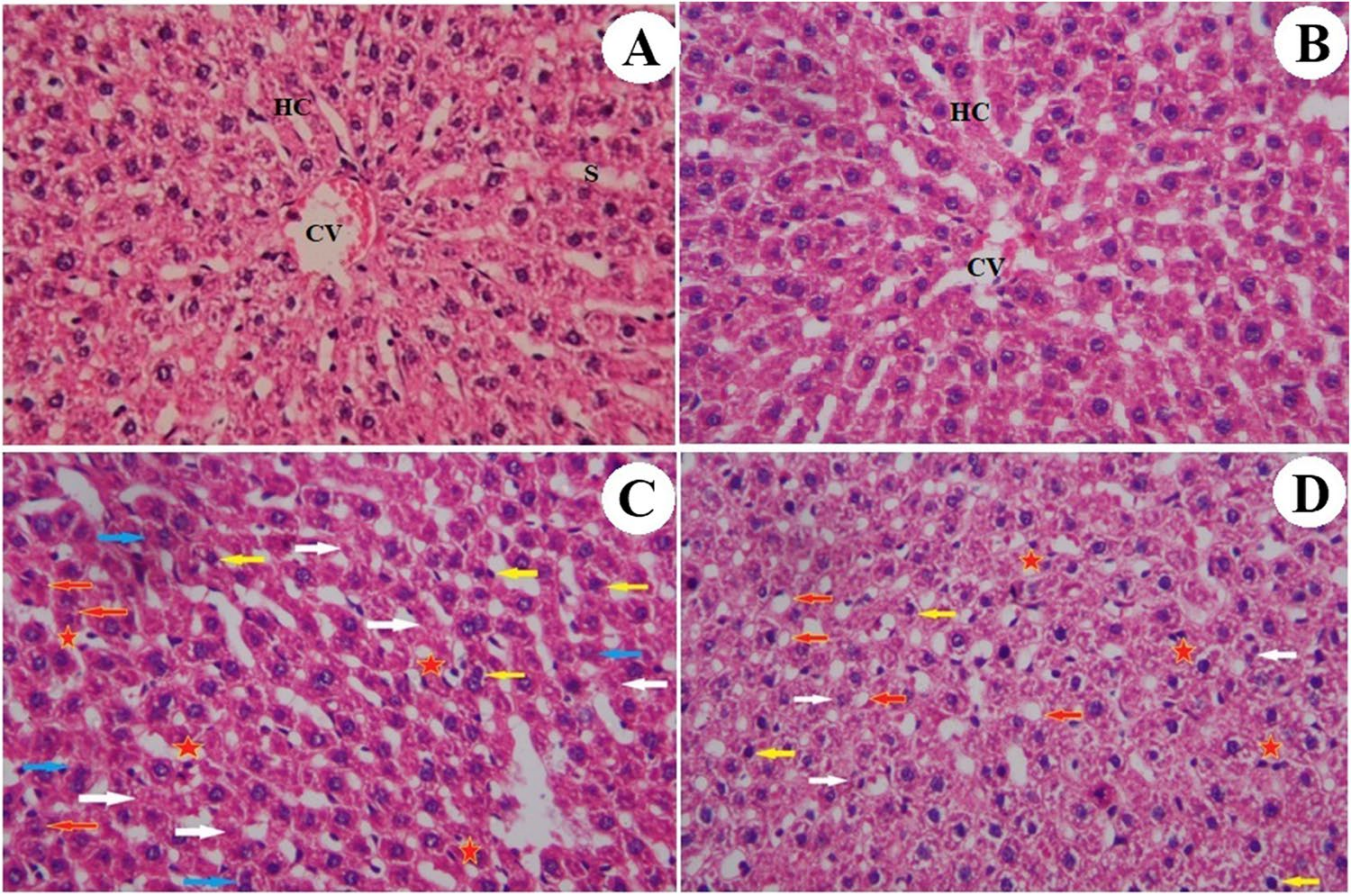
**A**, **B** A photomicrograph of a section of control and NAC livers showing the architecture of a hepatic lobule. The central vein (CV) lies at the center of the lobule surrounded by the hepatocytes (HC) with strongly eosinophilic granulated cytoplasm, and distinct nuclei. Between the strands of hepatocytes, the hepatic sinusoids (S) are shown. **C** A photomicrograph of a section of the liver of the CdCl_2_ group showing the disturbance of the hepatic lobule. Notice the presence of apoptotic (red arrows) and necrotic (blue arrows) hepatocytes, infiltration of inflammatory cells (red star), hydropic degeneration (white arrows), and pyknotic nuclei (yellow arrows). **D** A photomicrograph of a section of the liver of a rat administrated with cadmium and N-acetylcysteine showing improvement in the structure of the hepatic lobule. However, there are some apoptotic hepatocytes (white arrows), pyknotic nuclei (yellow arrows), infiltration of inflammatory cells (red star), and fat deposition (red arrows) (H&E stain, magnification = 400 ×)

Figure 7A shows representative hematoxylin and eosin staining of normal rat liver tissue. Regarding the administration of NAC alone for 60 days, the hepatic lobules appeared more or less as the control one. The CdCl_2_ group showed disturbance of the hepatic lobule, presence of hepatocyte focal necrosis, hydropic degeneration, and pyknotic nuclei (Fig. 7B). In case of post-administration of NAC to CdCl_2_, the hepatic lobules appeared more or less as control rats (Fig. 7C), although some sections showed dilated portal tract associated with congested blood vessels and necrotic hepatocytes. This can be attributed to the antioxidant and free radical quenching efficacy of NAC. The scoring summary of the liver histopathological alterations was presented as a supplementary data (Table S1).

## Discussion

Oxidative stress plays a major role in Cd-induced organ toxicity and carcinogenicity (Rafati Rahimzadeh et al. 2017; Cuypers et al. 2010). Cadmium incidentally reasons ROS generation and subsequently oxidative damage of lipids, proteins, and DNA (Matovic et al. 2015; Yang and Shu 2015). These deleterious effects occurred via its inhibitory effect on the antioxidant defense system (enzymatic and non-enzymatic antioxidants) (Thevenod and Lee 2013; Matovic et al. 2011).

The present study firmly resolved many aspects, such as DNA content, cell cycle progression phases, ratio of anti-apoptotic Bcl‐2 to pro-apoptotic Bax, biochemical changes, and correlated histological changes to evaluate the therapeutic potential of NAC against Cd-induced toxicity. CdCl_2_ could shift the cell cycle from *G*_0_/*G*_1_ to *S* and *G*_2_/*M* phases and trigger the *S* and *G*_2_/*M* phase cell cycle arrest. Post-administration of NAC to CdCl_2_ showed a significant decrease in cell accumulation in *S* and *G*_2_/*M* phases compared to the CdCl_2_ group. The present results are in concordance with Odewumi et al. (2011) and Kim et al. (2005). No significant changes were noted in pro-apoptotic *Bax* and *Casp3* in the CdCl_2_ group. However, a significant increase in *Bcl2* and *Bcl2/Bax* ratio in the CdCl_2_-treated group suggests the tendency of hepatocytes toward resisting apoptosis. These results are in agreement with the previous studies on Cd-induced genotoxicity and carcinogenicity (Bjorklund et al. 2019; Buha et al. 2018). Post-administration of NAC to CdCl_2_ triggered the apoptotic pathway through a slight elevation in the levels of Bax and Cas-3 and a significant decrease in the Bcl2/Bax ratio when compared to the CdCl_2_ group.

Two signs were reported to indicate Cd-induced hepatotoxicity: the first one is the boosted activity of liver enzyme biomarkers, while the second is the historical tracking of liver structure changes. The results of the present study were found to be corresponding with a previous study carried out by Ramaiah (2007). The study shows that the increased circulating liver marker enzymes are an accurate sign to determine the extent of hepatic damage (Ramaiah 2007). When detailing the results in comparison to the control group, a noticeable change was found in liver function enzymes which would be indicated by increased levels in ALT and AST and a decrease in ALP. In the conducted study, NAC significantly improved the circulating levels of hepatic enzymes and markedly improved the histological structure of the liver, suggesting membrane stabilizing and potent hepatoprotective potential of NAC.

Several key results of CdCl_2_ treatment matched several previously published data, among which is the marked increase in NO, reacting with superoxide, forming hydroxyl radical and peroxynitrite which is a potent oxidant. The second notable result is the cellular GSH levels diminishing, while the last key result concluded a substantial increase in LPO (L’Hoste et al. 2009; Nair et al. 2015). Hence, it is safe to conclude that oxidative stress and lipid peroxidation are commonly acknowledged in the pathogenesis of various diseases. Another key conclusion is that the role of free radical scavenging antioxidants managed to gain attention and increase in reputation.

Reduced glutathione (GSH) is involved in maintaining the balance of cellular redox processes (Lushchak 2012) facilitating the production of ROS which lead to oxidative stress and increased lipid peroxidation (Clement et al. 2018). After NAC post-treatment, there were a significant decline in LPO and NO expression level (Zhang et al. 2018) and a significant increase in GSH (Correa et al. 2011) when compared to the CdCl_2_ group. GSH levels, which resulted in less oxidative stress and attenuated cell necrosis, are in concordance with published data (Mahmoud et al. 2019; Wang et al. 2020). The antioxidant enzyme activities (SOD, CAT, GPx) were decreased significantly in the CdCl_2_ group when compared with the control group and these data agree with previous studies (Mumtaz et al. 2020; Almeer et al. 2018; Ezedom et al. 2020). Meanwhile, adding NAC to CdCl_2_ resulted in a significant increase in the activity of SOD, CAT, and GPx enzymes when compared to their level in the CdCl_2_ group. These are consistent with Samuni et al. (2013) and Atagana and Asagba (2014). These enzymes are the primary antioxidant enzymes of the endogenous defense systems that protect cells from oxidative damage and exposure to cadmium causes inhibition of them. Cd can generate free radicals through decreasing the activities of antioxidant enzymes such as CAT, GPx, and SOD, or intracellular levels of antioxidants such as glutathione (Chen and Shaikh 2009).

The increased levels of these antioxidant enzymes with post-treatment of NAC could play a role to scavenge the oxygen radicals produced in the liver, which are in agreement with previous studies (El-Tarras Ael et al. 2016; Rani et al. 2014; Singh et al. 2012). Adding NAC to CdCl_2_ significantly increased tissue SOD, CAT, and GPx activities which are consistent with Esrefoglu et al. (2006) and Samuni et al. (2013).

CdCl_2_ induced liver injury involving oxidative and nitrosative stresses potentiating the damage to the liver tissues as validated by our histological results. The liver tissue in the CdCl_2_ group showed a disturbance of the hepatic lobule, presence of focal necrosis of the hepatocytes, hydropic degeneration, and pyknotic nuclei. Meanwhile, the post-treatment with NAC attenuates the functional deterioration of the cells and reduces cell necrosis and our results are supported by previously published data (Skvarc et al. 2017; Cusumano et al. 2015; Sangsefidi et al. 2020; Esrefoglu et al. 2006; Abdel-Daim et al. 2019). In the current study, the histopathological changes are correlated with the biochemical changes.

### Study limitations

Limitations of the present study were the dose-dependent effect that is not evaluated and the short duration of the study which might limit the outcomes precisely. Despite these limitations, this experimental study illustrated the carcinogenicity of cadmium, which is linked to the initiation of rodent cancer in the liver by defecting the oxidant/antioxidant balance, cell cycle, and programmed cell death pathway. However, the findings of this study are likely to translate to other species or systems, including human cancers.

## Conclusion

In conclusion, the present results clearly validate the effectiveness of NAC in protecting rat liver against oxidative stress resulting from CdCl_2_ intoxication. This is followed by a rising in GSH content as well as the SOD activity, while decreasing the LPO and NO levels. Decreased LPO suggests membrane firmness, which correlates with the increased activity of SOD. The mechanism by which NAC applies its antioxidant activity may be explained throughout stimulating the expression of a range of phase II antioxidant defense genes including the two subunits that control GSH synthesis. In addition, NAC opposes proliferation activity, lowering Bcl2/Bax ratio and upregulating *Casp3*, which leads to apoptotic fate.

## Supplementary Information

Below is the link to the electronic supplementary material.

Supplementary file1 (DOCX 12 KB)

## Data Availability

All relevant data are within the paper.
