# Supplementary material for: Antioxidant capacity of N-acetylcysteine against the molecular and cytotoxic implications of cadmium chloride leading to hepatotoxicity and vital progression
Source: Environ Sci Pollut Res Int. 2022 Nov 2;30(9):23237–47. doi: 10.1007/s11356-022-23823-x (PMC9938820; doi:10.1007/s11356-022-23823-x)
Supplement: Supplementary file 1 — Supplementary file1 (DOCX 12 KB) [file 11356_2022_23823_MOESM1_ESM.docx]

**Supplementary data; Table S1:** A summary of the liver histopathological alterations among the different groups.

|  | Control | NAC | CdCl_2_ | NAC+CdCl_2_ |
| --- | --- | --- | --- | --- |
| Hepatocytes degeneration | - | - | +++ | + |
| Congested central vein | - | - | ++ | + |
| Inflammatory cells infiltration | - | - | ++ | ++ |
| Apoptosis and necrosis | - | - | ++ | + |

−: no; +: mild; ++: moderate; +++: severe histological alterations.
